# Functional kombucha production from fusions of black tea and Indian gooseberry (*Phyllanthus emblica* L.)

**DOI:** 10.1016/j.heliyon.2024.e40939

**Published:** 2024-12-06

**Authors:** Haruthairat Kitwetcharoen, Yupaporn Phannarangsee, Preekamol Klanrit, Sudarat Thanonkeo, Patcharaporn Tippayawat, Poramaporn Klanrit, Poramate Klanrit, Mamoru Yamada, Pornthap Thanonkeo

**Affiliations:** a Department of Biotechnology, Faculty of Technology, Khon Kaen University, Khon Kaen, 40002, Thailand; b Fermentation Research Center for Value Added Agricultural Products (FerVAAP), Khon Kaen University, Khon Kaen, 40002, Thailand; c Walai Rukhavej Botanical Research Institute (WRBRI), Mahasarakham University, Maha Sarakham, 44150, Thailand; d Faculty of Associated Medical Sciences, Khon Kaen University, Khon Kaen, 40002, Thailand; e Research Group of Chronic Inflammatory Oral Diseases and Systemic Diseases Associated with Oral Health, Department of Oral Biomedical Sciences, Faculty of Dentistry, Khon Kaen University, Khon Kaen, 40002, Thailand; f Department of System Biosciences and Computational Medicine, Faculty of Medicine, Khon Kaen University, Khon Kaen, 40002, Thailand; g Department of Biological Chemistry, Faculty of Agriculture, Yamaguchi University, Yamaguchi, 753-8515, Japan; h Research Center for Thermotolerant Microbial Resources, Yamaguchi University, Yamaguchi, 753-8515, Japan

**Keywords:** Antioxidant, Antimicrobial, Black tea, Kombucha, Indian gooseberry fruits, SCOBY

## Abstract

The use of alternative ingredients as supplements to or blends with kombucha tea to improve organoleptic properties and health effects has recently increased. Indian gooseberry fruit is among the most promising alternative raw materials for producing functional kombucha since the berries contain several beneficial substances. In this study, the production of functional kombucha beverages from fusions of black tea and Indian gooseberry fruit homogenate (IGH) was investigated, and the chemical and biological properties of kombucha products were evaluated and compared with those of traditional black tea kombucha products. Chemical composition analysis revealed that IGH contains high amounts of polyphenols (627.4 mg GAE/L or 129.51 mg GAE/g dry weight), flavonoids (98.0 mg QE/L or 9.11 mg QE/g dry weight), and vitamins, specifically ascorbic acid (465.72 mg/100 g fresh weight). It also contains several amino acids, fatty acids, and trace elements. Supplementing or blending black tea kombucha with IGH in the range of 10 %–50 % (v/v) increased the total phenolic content (TPC), total flavonoid content (TFC), and total acidity of the fermented beverages. Several volatile organic compounds associated with the flavor, aroma, and health benefits of kombucha were also detected in black tea and IGH fusion kombucha products. Moreover, the black tea and IGH fusion kombucha products also displayed greater antioxidant and antimicrobial activities than the traditional black tea kombucha. Among the different combinations of black tea and IGH, supplementing black tea kombucha with 20 % IGH was the best combination for producing alternative and unique functional kombucha products with notable nutritional and health benefits.

## 1 Introduction

Due to their perceived health benefits, growing health concerns, and significant contribution to health promotion, functional beverages are considered one of the largest and fastest-growing segments of the functional foods sector [1]. Several functional beverages have been recently developed, and kombucha is a traditional, fermented, functional beverage produced and consumed globally [[2], [3], [4]]. Based on the high demand for functional beverages, the global market size of kombucha products is expected to reach $4.5 billion by 2025 [4]. Kombucha contains a variety of beneficial substances that are not only extracted from raw materials but also generated during fermentation; these substances include polyphenols, flavones, organic acids, vitamins, amino acids, trace elements, proteins, and hydrolytic enzymes [[5], [6], [7], [8]]. These valuable compounds possess many significant health effects and therapeutic properties, including antioxidant, antimicrobial, anti-inflammatory, and anticancer effects. Furthermore, kombucha can also lower blood pressure, promote liver function, enhance the immune system, and prevent several diseases, such as diabetes, hypertension, and cardiovascular diseases [3,4,7,[9], [10], [11], [12]].

Kombucha is typically produced from fusions of tea (*Camellia sinensis*) (commonly black and green teas) and sugar using a symbiotic culture of bacteria and yeast (SCOBY), which is also known as mushroom tea or tea fungus [4,5]. As previously reported, the content of beneficial substances and the biological properties of kombucha products vary depending on the type of raw material, source and microbial community of the SCOBY, and fermentation conditions [4,7]. For instance, Cardoso et al. [13] demonstrated that black tea kombucha exhibited significantly greater antioxidant activity than green tea kombucha because black tea contains a greater variety of polyphenols than green tea. Kaewkod et al. [14] and Jakubczyk et al. [15] noted that black tea kombucha contains more organic acids than green and oolong tea kombuchas. Conventional kombucha production is primarily based on tea leaves and sugar. However, due to consumer perceptions and the demand for healthy foods and beverages, functional kombucha products have recently been produced not only from tea leaves but also from other alternative ingredients, such as fruits, herbs, vegetables, honey, juices, and plant infusions, as supplements to or blends with kombucha tea [7,[16], [17], [18], [19], [20], [21]]. In addition to providing novel functional kombucha products, these alternative raw materials also enhance organoleptic properties and improve the beneficial health effects of kombucha [6,[22], [23], [24], [25]].

The Indian gooseberry fruit (*Phyllanthus emblica* L.), also known as Amla, Emblic myrobalan, Myrobalan, Malacca tree, and Makham Pom, is a plant native to southeastern Asia and is widely found in many regions, such as India, China, Sri Lanka, Pakistan, Bangladesh, Malaysia, and Thailand. This plant belongs to the Phyllanthaceae family and is commonly used in Indian Ayurvedic medicine to treat diarrhea, jaundice, inflammation, and many other ailments [[26], [27], [28], [29]]. Indian gooseberry fruit is highly nutritious and is among the richest sources of ascorbic acid or vitamin C; the vitamin C content is 200–900 mg/100 g of the edible portion, which is significantly greater than that found in oranges, tangerines, and lemons. Gooseberries also contain various other beneficial compounds, including tannins, alkaloids, flavones, glycosides, organic acids, amino acids, trace elements, and polyphenols [[28], [29], [30], [31]], specifically in the fruiting body. Based on previous *in vitro* studies, Indian gooseberry fruits are known to have a variety of health benefits, such as antibacterial, antifungal, antiviral, antioxidant, anti-inflammatory, antidiabetic, anticancer, antiulcerogenic, and other therapeutic properties, such as gastroprotective, hepatoprotective, hypolipidemic, and chemopreventive effects [28,[32], [33], [34]]. In animal research, Indian gooseberry extract is effective in the management of dyslipidemia by reducing low-density lipoprotein (LDL) cholesterol and increasing high-density lipoprotein (HDL) cholesterol levels in experimental rabbits [35]. In rat models, the gooseberry extract also exhibits antidiabetic potential and hypoglycemic effects [36,37], as well as analgesic effects in postoperative and neuropathic pain models [38]. Furthermore, the protective and therapeutic effects of Indian gooseberry extract have also been studied clinically. The plant extract significantly reduces total cholesterol, LDL, triglycerides, hyperglycemia and dyslipidemia and significantly enhances HDL levels in Type 2 diabetes mellitus (T2DM) patients [39,40]. The Indian gooseberry extract also exhibits potential hypoglycemia effects in dyslipidemia patients [41].

Based on the literature review, a few studies have been reported on kombucha production using Indian gooseberry fruits as an alternative substrate. Indeed, only green tea kombucha production supplemented with Indian gooseberry fruits has been investigated [6,42]. Thus, this study aimed to develop functional kombucha beverages from fusions of black tea and Indian gooseberry fruits. The chemical compositions and biological properties of the functional kombucha products were also evaluated.

## 2 Materials and methods

### 2.1 Chemical and plant materials

Glucose was obtained from KemAusTM, Australia. Ethanol, glacial acetic acid, gallic acid, quercetin, 2,4,6-tris(2-pyridyl)-s-triazine (TPTZ), 2,2−diphenyl−2−picrylhydrazyl (DPPH), and 2,2’−azino−bis−(3−ethylbenzothiazoline−6−sulfonic acid) (ABTS) were purchased from Sigma−Aldrich (St. Louis, MO, USA). All the other chemicals were procured from a local supplier (CLS Supply and Services, Ltd. Part., Khon Kaen, Thailand).

Dried black tea leaves were obtained from the Royal Project Tea Highland, Chiang Mai, Thailand. Indian gooseberry fruits were purchased from local markets in Khon Kaen, Chaiyaphum, and Roi Et provinces, Thailand. The fruits were washed thoroughly with running tap water, the seeds were removed, and the resulting fruit pulps were ground using a fruit blender (HR2225/00, Philips, China). The resulting Indian gooseberry fruit homogenate (IGH) was kept at −20 °C before use. The phytochemical parameters in the IGH, such as the total sugar, vitamin, amino acid, fatty acid, and trace element contents, total phenolic content (TPC), total flavonoid content (TFC), and antioxidant activity, were determined.

### 2.2 Preparation of kombucha starter or kombucha SCOBY

Sweetened black tea was prepared by soaking 4.5 g of dried tea leaves in 1 L of boiling water for 10 min. After removing tea leaves by filtration, 60 g of sucrose was added, and the mixture was stirred until the sugar completely dissolved. The resulting sweetened black tea was filled in sterile glass jars, and 5 % (v/v) of mother kombucha SCOBY (Chiira Organic, Bangkok, Thailand) was added. After statically incubating at 25 °C for 14 days, the new daughter kombucha SCOBY was obtained and used as a kombucha starter for the subsequent experiments.

### 2.3 Production of kombucha beverages from fusions of black tea and IGH

**Fig. 1:**
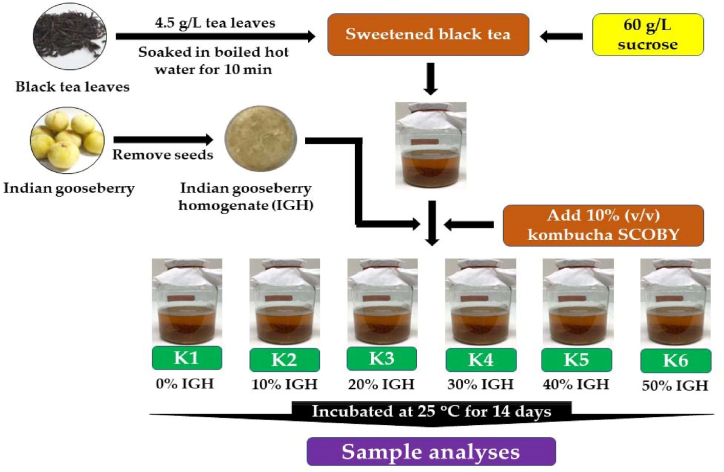
The flow diagram for the preparation of kombucha beverages made from fusions of black tea and IGH.

The production of kombucha beverages from fusions of black tea and IGH was performed using the production guidelines of Jayabalan et al. [5] and Phung et al. [7], with some modifications. The flow diagram for the production process of kombucha beverages from fusions of black tea and IGH is illustrated in Fig. 1. Sweetened black tea was prepared using the same procedure described in Section 2.2. Based on the preliminary study, a high concentration of IGH, specifically at a concentration greater than 50 % (v/v), suppressed the growth of the microbial community in the kombucha SCOBY due to high total acidity with a relatively low pH level (pH of 2.5). Thus, IGH concentrations ranging from 10 % to 50 % (v/v) were used in this study. Traditional black tea kombucha without supplementation of IGH was used as a control treatment (K1). The black tea and IGH fusions kombucha beverages were made by adding different concentrations of IGH into sweetened black tea, i.e., 10 % (K2), 20 % (K3), 30 % (K4), 40 % (K5), and 50 % (v/v) IGH (K6). All the kombucha treatments were transferred into a glass jar, and 10 % of the kombucha starter was added. The fermentation jars were statically incubated at 25 °C for 14 days. Kombucha samples were randomly collected every two days, after which the chemical compositions and biological properties of the kombucha products were analyzed.

### 2.4 Determination of volatile organic compounds using solid-phase microextraction (SPME)-gas chromatography-mass spectrometry (GC-MS)

Volatile organic compounds in the kombucha products were determined using the procedure described by Tejedor-Calvo et al. [43]. The aromatic compounds were extracted from the kombucha samples by SPME using a fiber coated with a 50/30 μm layer of divinyl benzene/carboxen/polydimethylsiloxane. Three milliliters of each sample was transferred into the glass vial and then incubated at 60 °C for 10 min. After that, the fiber was exposed to the headspace of the vial for 30 min. The profile of volatile organic compounds from each sample was analyzed by GC (Agilent 7890A) and a mass spectrometry detector (Agilent 7000B, Agilent Technologies, Inc., Palo Alto, CA, USA) using a DB-wax capillary column (length, 60 m; diameter, 0.25 mm; film thickness, 0.25 μm). Helium was used as a carrier gas at a 1 mL/min flow rate. The injection and transfer line temperature was set up at 250 °C. The temperature in the column oven was held at 35 °C and increased from 35 to 180 °C at a rate of 3 °C/min and finally to 230 °C at a rate of 10 °C/min and held for 10 min. Electron ionization (EI) with an energy potential of 70 eV and ion source temperature of 230 °C were applied for MS determination. Full scan mode (35−500 *m*/*z*) for MS scanning and GC-QQQ software for controlling the system were employed.

Identification and semi-quantification of the volatile organic compounds were carried out based on the method described by Tejedor-Calvo and Morales [44]. The peak identification of volatile organic compounds was assessed by comparing the mass spectra of the samples with mass spectral data from the NIST MS Search Version 2.0 library, which was carried out at the Center of Scientific and Technological Equipment, Suranaree University of Technology, Thailand. The content of volatile organic compounds (semi-quantification) was expressed as a relative percentage area, which was determined by integrating the area of the sum of ion characteristics of each compound and calculating the relative percentage.

### 2.5 Measurement of the antioxidant activity of kombucha products

#### 2.5.1 Ferric reducing antioxidant power (FRAP) assay

The antioxidant potential of kombucha products for reducing Fe^3+^ to Fe^2+^ was determined using the ferric reducing antioxidant power (FRAP) assay [45]. The FRAP reaction was prepared by mixing 50 μL of kombucha product, 1.5 mL of FRAP reagent (300 mM acetate buffer (pH 3.6), 10 mM 2,4,6-tris(2-pyridyl)-s-triazine (TPTZ), 20 mM ferric chloride), and 150 μL of distilled water. After 10 min of incubation in the dark at room temperature, the absorbance was measured at a wavelength of 593 nm. The antioxidant activity was calculated based on the standard curve of ferrous sulfate heptahydrate and expressed as g Fe(II)/L of kombucha products.

#### 2.5.2 2,2-Diphenyl-2-picrylhydrazyl (DPPH) radical-scavenging activity

The free radical scavenging activity of the kombucha products was determined by the DPPH assay using the protocol of Chan et al. [46] with slight modifications. Briefly, 0.2 mL of the kombucha sample was added to 3 mL of 0.1 mM DPPH solution and vigorously mixed using a vortex mixer. After incubating in the dark at room temperature for 30 min, the absorbance of the reaction mixture was measured at a wavelength of 517 nm. The percentage of DPPH radical scavenging activity was calculated using Equation (1).

(1) % DPPH radical scavenging activity = [(A_control_ − A_sample_)/A_control_] × 100

where A_control_ is the reaction mixture without sample solution, and A_sample_ is the reaction mixture with the sample solution.

#### 2.5.3 2,2’−azino−bis(3−ethylbenzothiazoline−6−sulfonic acid) (ABTS) radical scavenging assay

The radical ABTS^+^ scavenging activity was evaluated using the method of Mukherjee et al. [47] with some modifications. Briefly, 100 μL of each dilute kombucha sample was added to 3.8 mL of ABTS•^+^ reaction solution, which was prepared by mixing 7 mM ABTS stock solution with 2.45 mM potassium persulfate. After incubating in the dark at room temperature for 6 min, the absorbance was measured at a wavelength of 734 nm. The percentage of ABTS^+^ inhibition was calculated from Equation (2).

(2) % ABTS^+^ inhibition = [(A_control_ − A_sample_)/(A_control_)] × 100

where A_control_ is the reaction mixture without sample solution, and A_sample_ is the reaction mixture with the sample solution.

### 2.6 Determination of the antimicrobial activity of kombucha products

The antimicrobial activity of the kombucha products was assessed using the agar well diffusion method [14] with slight modifications. Gram-negative bacteria (*Enterobacter cloacae*, *Escherichia coli* ATCC 25922, *Pseudomonas aeruginosa* ATCC 27853, *Salmonella enterica* Enteritidis, *S. enterica* Typhimurium, *S. enterica* Typhi DMST 22842, and *Shigella dysenteriae* DMST 1511), gram-positive bacteria (*Bacillus cereus*, *Listeria monocytogenes* ATCC 19115, and *Staphylococcus aureus* ATCC 25923), and yeast (*Candida albicans*) kindly provided by the Faculty of Associated Medical Science, Khon Kaen University, were used in this study. These pathogenic microorganisms were cultured in brain heart infusion (BHI) broth and incubated in a rotary incubator shaker at 37 °C and 150 rpm. After 18 h of cultivation, the cells were removed and spread on Mueller–Hinton (MH) agar plates. Agar wells with a 6 mm diameter were made using a sterile cork borer, and kombucha samples were collected after centrifugation at 10,000 rpm for 5 min and filtration through a 0.22 μm sterile microfilter. One hundred microliters of resulting kombucha samples was added into agar wells. After being kept at 4 °C for 2 h, the plates were subsequently incubated at 37 °C for 24 h, and the zone of microbial growth inhibition was monitored according to Battikh et al. [48]. Acetic acid at 8.5 g/L and distilled water were used as positive and negative controls, respectively. They were applied into agar wells with a final volume of 100 μL [7].

### 2.7 Analytical procedures

Amino acids, fatty acids, and vitamins in the IGH were determined by high-performance liquid chromatography (HPLC) using a refractive index (RI) detector (Shimadzu, Kyoto, Japan) and an Aminex HPX-87H column (300 × 7.8 mm) (Bio-Rad, Hercules, CA, USA). Trace elements were examined using atomic absorption spectroscopy (AAS) at the Central Laboratory (Thailand) Co., Ltd., Khon Kaen branch, Thailand, while total sugar was analyzed using the phenol‒sulfuric acid method [49]. The pH values of the samples were evaluated by an electronic pH meter (FE28 FiveEasy, Mettler Toledo, Switzerland), while the total acidity was examined using the protocol described by Osiripun et al. [50]. TPC and TFC were assessed by the Folin–Ciocalteu method [15] and aluminum chloride colorimetric assay [51], respectively, using gallic acid and quercetin as standards. The TPC and TFC are reported as milligrams of gallic acid equivalents (GAE) per liter of the sample (mg GAE/L) and milligrams of quercetin equivalent (QE) per liter of the sample (mg QE/L), respectively. The ethanol content in the kombucha products was examined by gas chromatography (GC) (GC-14B, Shimadzu, Kyoto, Japan), and the analytical instrument was equipped with a polyethylene glycol (PEG-20 M)-packed column and a flame ionization detector (FID). Kombucha color was evaluated based on the L∗, a∗, and b∗ values using an Ultrascan XE SN-U3115 (Hunterlab, Reston, VA, USA) instrument with a standard reference method.

### 2.8 Statistical analysis

The design used for the kombucha experiments was based on a completely randomized design (CRD) with experiments conducted in triplicate. The experimental data were statistically analyzed using one-way analysis of variance (ANOVA) at a probability of p ≤ 0.05. The significant differences between the treatments were determined based on Duncan's multiple range test (DMRT) using SPSS version 28.0 (IBM SPSS Statistics 28, IBM Corporation, Armonk, NY, USA). All the data are presented as the means ± standard deviations (SDs).

## 3 Results and discussion

### 3.1 Major components and antioxidant activity of the raw materials

It has been reported that black tea and Indian gooseberry fruits contain various beneficial components, including polyphenols and flavonoids [3,5,28,33,34]. As shown in this study, black tea extract contained 1124.8 mg GAE/L polyphenols, which was approximately 1.8-fold higher than that detected in Indian gooseberry fruit juice (627.4 mg GAE/L). The TPC of black tea detected in the present study based on the dry matter was in the range reported by Dufresne and Farnworth [52] but lower than that reported by KC et al. [53], whereas the TPC of Indian gooseberry fruits was similar to that reported in the study of Poltanov et al. [54] but significantly greater than that reported by Pareek et al. [33] and Pawar [55]. The differences in the TPC of Indian gooseberry fruits might be due to differences in plant varieties, growth conditions, harvesting stages, and drying processes. The TFC of black tea extract was 90.2 mg QE/L, while that of Indian gooseberry fruit juice was 98.0 mg QE/L. The total flavonoid concentration in black tea (based on dry matter) used in this work was slightly lower than the value reported by Leal et al. [10], possibly due to differences in the plant varieties, harvesting stages, and manufacturing processes of the tea leaves. Notably, the TFC of Indian gooseberry fruits reported in this study was within the range reported by Poltanov et al. [54] and Pawar [55].

Because they contain a wide variety of beneficial compounds, black tea and Indian gooseberry fruits are considered good sources of a wide range of health-promoting substances, including those with antioxidant activity. As demonstrated in this research, the radical scavenging efficacy and free radical inhibition ability of black tea were 66.37 % and 23.28 mM Fe(II)/L, respectively, which were markedly lower than those detected in Indian gooseberry fruits (84.14 % inhibition and 49.60 mM Fe(II)/L). The high antioxidant activity of Indian gooseberry fruits compared with black tea might be correlated with a high ascorbic acid (vitamin C) content. The concentration of ascorbic acid in the Indian gooseberry fruits used in this research was 465.72 mg/100 g fresh weight (FW) (Table 1), which was within the range reported by Pareek et al. [33]. A study by Pawar [55] also reported a similar amount of ascorbic acid in the Indian gooseberry fruits (460 mg/100 g FW). In contrast, a high ascorbic acid content in Indian gooseberry fruits has also been reported. For instance, Chauhan et al. [56] noted that Indian gooseberry fruits contain ascorbic acid at concentrations ranging from 500 to 1500 mg/100 g FW. Kulkarni and Ghurghure [57] pointed out that Indian gooseberry fruits contain 600 mg/100 g FW of ascorbic acid, while Tewari et al. [58] stated that the concentrations of ascorbic acid in the Indian gooseberry fruits varied ranging from 498 to 585 mg/100 g FW. A recent report by Ikram et al. [59] mentioned that Indian gooseberry fruits contain ascorbic acid in the range of 470–680 mg/100 g FW, depending on the plant cultivars, plant sources, and ripening and harvesting stages.

**Table 1:** Phytochemicals in the Indian gooseberry fruits used in this research.

Main component	Chemical	Concentration (mg/100 g FW)
Vitamins	Ascorbic acid (Vit. C)	465.72
	Thiamine (B1)	0.04
	Niacin (B3)	0.01
	Pantothenic acid (B5)	0.06
Amino acids	Alanine	40.00
	Arginine	80.00
	Aspartic acid	50.00
	Glycine	30.00
	Isoleucine	20.00
	Leucine	40.00
	Methionine	90.00
	Phenylalanine	20.00
	Proline	50.00
	Threonine	20.00
	Tryptophan	0.02
	Valine	0.05
Fatty acids		
Saturated fatty acids	Butyric acid	95.50
	Caproic acid	19.80
	Undecanoic acid	13.50
	Palmitic acid	10.10
	Stearic acid	7.00
	Arachidic acid	4.50
	Behenic acid	1.30
Unsaturated fatty acids	Cis-9-oleic acid	4.10
	Cis-11-eicoseneoic acid	3.60
	Nervonic acid	1.70
	Cis-9,12-linolenic acid	5.40
	Cis-5,8,11,14,17-eicosapentaenoic acid	4.10
Trace elements	Calcium (Ca)	57.25
	Copper (Cu)	0.01
	Iron (Fe)	0.18
	Magnesium (Mg)	9.88
	Manganese (Mn)	0.20
	Nitrogen (N)	690.00
	Phosphorus (P)	13.80
	Potassium (K)	209.00
	Sodium (Na)	116.90
	Zinc (Zn)	0.06

In addition to ascorbic acid, Indian gooseberry fruits also contain other vitamins, specifically vitamin B, a water-soluble vitamin that is known to be an essential cofactor for several enzymes involved in metabolic pathways, such as purine and pyrimidine biosynthetic pathways or amino acid interconversion pathways. Vitamin B, specifically B3, is also known to promote human health by lowering cholesterol levels and improving blood circulation [60,61]. As shown in Table 1, pantothenic acid or vitamin B5 was the most common vitamin in Indian gooseberry fruits, with a content of 0.05 mg/100 g FW, followed by thiamine or vitamin B1 (0.04 mg/100 g FW) and niacin or vitamin B3 (0.004 mg/100 g FW). Other vitamins were not detected in this study. Notably, the B1, B3, and B5 concentrations observed in the present study aligned with those values reported by Pareek et al. [33]. However, the content of vitamin B3 in this study was remarkably lower than that reported by Kulkarni and Ghurghure [57].

As previously reported by Srivasuki [31], Indian gooseberry fruits are rich not only in ascorbic acid but also in amino acids and trace elements, which play essential roles in microbial growth and metabolism. Specifically, amino acids are also involved in the biogenesis of the aroma of kombucha [52]. As shown in Table 1, various amino acids were detected in the Indian gooseberry fruits used in this research, in accordance with that reported by Tewari et al. [62]. Methionine was the primary amino acid found in Indian gooseberry fruits, yielding 90 mg/100 g FW, followed by arginine (80 mg/100 g FW), aspartic acid (50 mg/100 g FW), and proline (50 mg/100 g FW). The lowest amino acids detected in Indian gooseberry fruits were tryptophan (0.02 mg/100 g FW) and valine (0.05 mg/100 g FW). The concentrations of amino acids found in gooseberry fruits in this work differ from those reported by Pareek et al. [33], possibly due to differences in plant varieties, plant sources, harvesting processes, and detection methods. Considering the trace elements, nitrogen (N) was the major element found in the raw material, with a concentration of 690.0 mg/100 g FW, followed by potassium (K) (209.0 mg/100 g FW) and calcium (Ca) (57.3 mg/100 g FW). Copper (Cu) and zinc (Zn) yielded the least abundant trace elements, with concentrations of 0.01 and 0.06 mg/100 g FW, respectively. Although the content of trace elements detected in the current study followed the findings of Pareek et al. [33], some elements, such as Ca and P, displayed remarkably higher concentrations than those reported by Kulkarni and Ghurghure [57].

In addition to polyphenols, flavonoids, vitamins, amino acids, and minerals, Indian gooseberry fruits also contain sugars and several fatty acids. The total sugar concentration in the Indian gooseberry fruits was approximately 8 g/L, a remarkably lower concentration than that reported by Tewari et al. [62]. Among the fatty acids identified, butyric acid emerged as the most abundant saturated fatty acid, with a concentration of 95.5 mg/100 g fresh weight (FW). In contrast, cis-9,12-linolenic acid was the predominant unsaturated fatty acid, present at 5.4 mg/100 g FW (Table 1). These fatty acids play a key role in kombucha production: contributing to aroma development during fermentation and offering potential health benefits. Notably, butyric acid stands out for its significant health-promoting properties. Research indicates that this fatty acid supports gut barrier function, reduces inflammation, and may potentially prevent colorectal cancer by promoting colonocyte health and modulating immune responses [[63], [64], [65]].

It should be noted in this study that Indian gooseberry fruits exhibited high total acidity (3.92 %) and a relatively low pH (pH 2.5), possibly due to the high content of organic acids, particularly ascorbic acid. On the other hand, black tea is mildly acidic and has a pH of 4.6. This mild acidity may be associated with the organic acids in tea leaves, such as tannic acid, malic acid, oxalic acid, citric acid, shikimic acid, and quinic acid [[66], [67], [68]]. Based on the wide variety and concentration of phytochemicals in Indian gooseberry fruit, it is considered one of the most promising alternative ingredients for supplementing or blending with tea leaves for functional kombucha production.

### 3.2 Biochemical changes during kombucha production

**Fig. 2:**
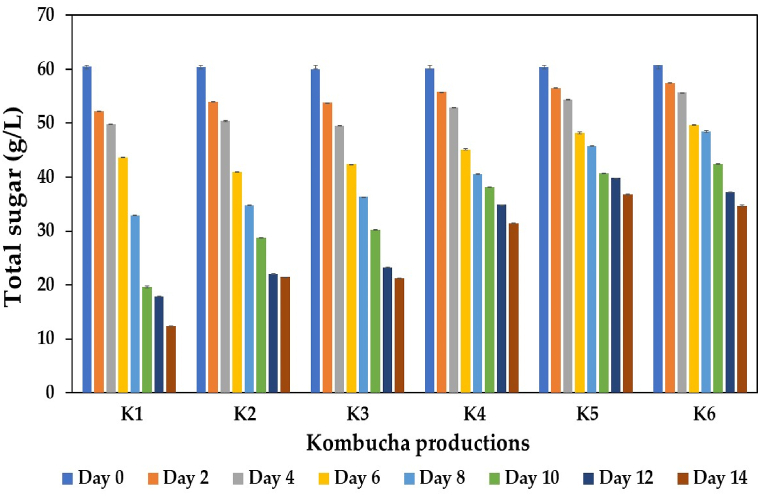
Changes in total sugar content during kombucha production from black tea alone or with IGH fusions. K1 is black tea kombucha, while K2 to K6 are black tea kombuchas supplemented with 10 %, 20 %, 30 %, 40 %, and 50 % IGH, respectively.

The biochemical changes during kombucha fermentation were monitored, including total sugar content, pH, TPC, TFC, ethanol content, and acidity. Generally, the initial sugar concentration for kombucha production ranges from 50 to 200 g/L [10]. In this work, the initial sugar concentrations of all the kombucha production formulations (K1 to K6) were adjusted to approximately 60 g/L to produce functional kombucha beverages. As shown in Fig. 2, a reduction in sugar concentration was observed during the fermentation process, and the sugar consumption rate varied depending on the amount of IGH added to the sweetened black tea. The control treatment without the addition of IGH (K1) and the treatments with 10 % (K2) and 20 % IGH supplementation (K3) resulted in a faster sugar consumption rate than the other treatments, possibly associated with the initial pH of the fermentation medium. The initial pH of the control treatment (K1) was approximately 4.5, while the values of the K2 and K3 treatments were 3.7 and 3.5, respectively, which were in the acceptable range for the growth of yeasts and bacteria in the kombucha SCOBY [7,69,70].

At the end of kombucha fermentation (14 days), the lowest sugar concentration was detected in K1, at 12.37 g/L, while those in K2 and K3 were 21.42 and 21.17 g/L, respectively. A high proportion of IGH seemed to reduce the sugar consumption efficacy of the microbial community in kombucha SCOBY, possibly due to the high total acidity of kombucha beverages because IGH contains high amounts of organic acids, specifically ascorbic acid and citric acid [33,[56], [57], [58], [59]]. It has been previously reported that high acidity conditions can inhibit microbial growth and metabolic activity, especially in yeasts, which play a crucial role in converting sugar into ethanol during kombucha fermentation [4]. Notably, after 14 days of fermentation, the final concentrations of total sugars detected in K4, K5, and K6 ranged from 31.40 to 36.71 g/L, implying that approximately 38–48 % of the sugar was consumed. These findings suggested that high proportions of IGH were unfavorable for microbial growth and metabolism.

**Fig. 3:**
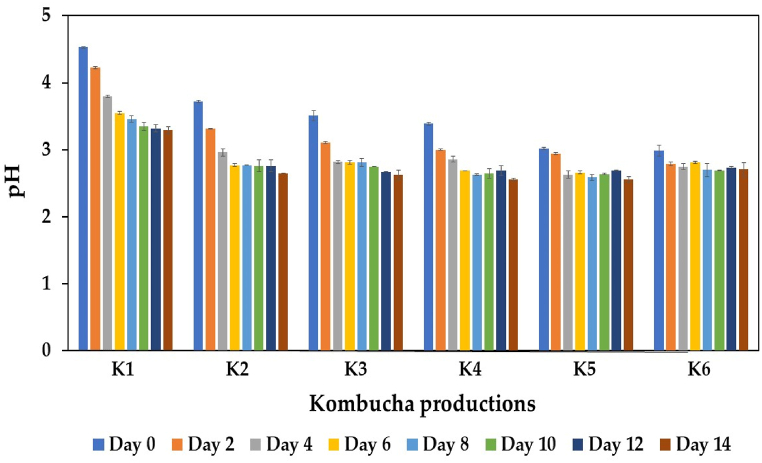
Changes in pH during kombucha production from black tea alone or with IGH fusions. K1 is black tea kombucha, while K2 to K6 are black tea kombuchas supplemented with 10 %, 20 %, 30 %, 40 %, and 50 % IGH, respectively.

The pH of kombucha also plays an essential role in fermentation, particularly in microbial growth, enzyme activity, and the stability of beneficial compounds in kombucha beverages [71]. The changes in pH during kombucha fermentation were monitored, and the results are shown in Fig. 3. The initial pH of the control kombucha (K1) was 4.5, slightly greater than that of the other treatments. Adding IGH reduced the initial pH of the kombucha beverages, especially at a high proportion of IGH supplementation, similar to the findings of Klawpiyapamornkun et al. [6]. One plausible explanation is that IGH contains high amounts of organic acids, particularly ascorbic and citric acids, causing a high total acidity of kombucha products at relatively low pH levels. The lowest initial pH (2.99) was detected in the K6 treatment supplemented with 50 % IGH. A reduction in the pH was observed in all the treatments during the fermentation of kombucha, and the final pH ranged from 3.29 to 2.56. Notably, kombucha produced from black tea and IGH fusions (K2 to K6) exhibited lower final pH values than kombucha produced from black tea alone (K1). Several organic acids, such as acetic acid, lactic acid, citric acid, gluconic acid, glucuronic acid, ascorbic acid, malic acid, oxalic acid, and butyric acid, are generated through the metabolic activity of the microbial community in kombucha SCOBY during kombucha fermentation [10]. Thus, the decrease in the kombucha pH might be attributed to the production and accumulation of these organic acids. The results of the present study aligned with those of Chakravotry et al. [72], Kaewkod et al. [14], Jakubczyk et al. [15], and Phung et al. [7], who reported a reduction in pH during kombucha production. In the present study, the final pH of all the kombucha beverages produced from black tea and IGH fusions was below 3.0. According to the Food and Drug Administration (FDA), the lowest acceptable pH for kombucha products is 3.0 [4]. To ensure product safety and compliance while maintaining functional properties, pH adjustment can be performed through careful blending with tea water. This approach allows for precise pH control without significantly compromising the beneficial microbial characteristics and bioactive compounds inherent to kombucha.

Polyphenols are among the most abundant beneficial compounds in kombucha products, and these substances play a significant role in preventing many diseases related to oxidative stress, such as cancer and cardiovascular and neurodegenerative diseases [73]. Furthermore, these compounds are also involved in cholesterol metabolism and prevent high blood pressure by promoting smooth muscle relaxation [74]. Polyphenols in kombucha beverages are not only extracted from raw materials but also generated during fermentation. The quantity of these compounds varies depending on several parameters, such as the microbial composition in kombucha SCOBY, the raw materials used, and fermentation conditions [10]. A study by Cardoso et al. [13] comprehensively identified a total of 127 phenolic compounds in green and black tea kombuchas. The compositional analysis revealed that flavonoids were the most prevalent, accounting for 70.2 % of the compounds, followed by phenolic acids at 18.3 %, other polyphenols at 8.4 %, lignans at 2.3 %, and stilbenes at 0.8 %. The most abundant phenolic compounds in green and black tea kombuchas included 5-O-Galloylquinic acid, quercetin 3-O-rhamnosyl-rhamnosyl-glucoside isomer 2, quercetin 3-O-glucosyl-rhamnosyl-galactoside isomer 2, quercetin 3-Ο-rhamnosyl-rhamnosyl-glucoside isomer 1, quercetin 3-Ο-glucosyl-rhamnosyl-galactoside isomer 1, 3-[2-(carboxymethyl)-3,4-dihydroxyphenyl]prop-2-enoic acid, 4-Coumaroylquinic acid isomer 2, and 1-O-Caffeoylquinic acid isomer 2/3-Caffeoylquinic acid. This diverse array of compounds underscores the complex chemical composition of kombucha and its potential health-promoting properties.

**Fig. 4:**
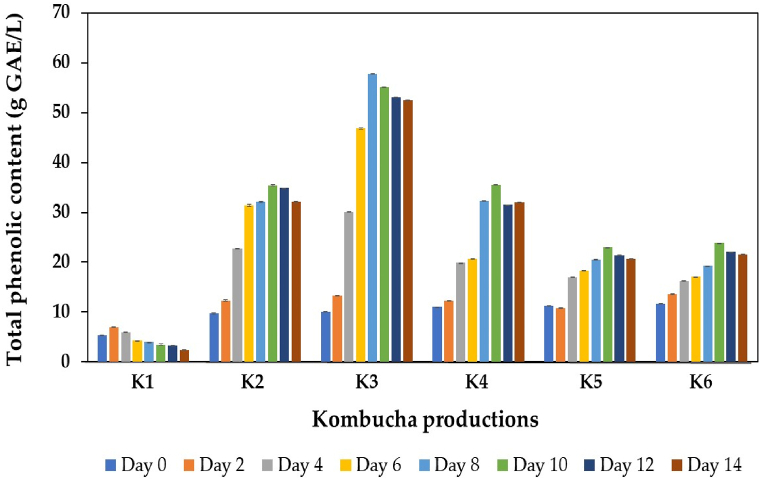
Changes in TPC during kombucha production from black tea alone or with IGH fusions. K1 is black tea kombucha, while K2 to K6 are black tea kombuchas supplemented with 10 %, 20 %, 30 %, 40 %, and 50 % IGH, respectively.

In this study, the changes in TPC during kombucha fermentation were examined, and the results are summarized in Fig. 4. The initial TPC of the control (traditional) kombucha (K1) was 5.31 g GAE/L, markedly lower than that produced from black tea and IGH fusions. The addition of IGH significantly increased the initial TPC of kombucha products approximately 2-fold, which might be attributed to the high polyphenol content in IGH [33,54,55]. The TPC of kombucha samples produced from black tea and IGH fusions tended to increase during kombucha production and slightly decreased at the end of fermentation compared with that of the control kombucha without IGH addition, which had a slightly lower TPC than that at the beginning of fermentation. The increase in TPC in black tea and IGH fusion kombuchas may be attributed to the release of polyphenols from Indian gooseberry fruits, similar to the findings of other studies in which black carrot [75], strawberry [24] and pineapple peel and core [7] were used as alternative raw materials for supplementation. In addition, the degradation of complex polyphenols in raw materials into small molecules by hydrolytic enzymes released from the microbial community in kombucha SCOBY may also contribute to the increase in TPC in kombucha beverages [72]. Furthermore, the beneficial metabolites generated during kombucha fermentation by the metabolic activity of microbial components, such as organic acids, ethanol, and esters, in kombucha SCOBY can also solubilize insoluble, bound polyphenols in plant cell walls, leading to an increase in the TPC in the finished products [76]. It should be noted in this study that among the kombucha samples produced from black tea and IGH fusions, the values of TPC during the fermentation process of K5 and K6 kombuchas are significantly lower than those of other kombucha samples (K2−K4). One possibility is that high proportions of IGH increased the total acidity of kombucha samples, which in turn caused a reduction in the microbial metabolic activity involved in the degradation of polyphenols in the raw materials, similar to a study using different proportions of pineapple peel and core as supplements with black tea kombuchas [7].

Notably, a reduction in the TPC of kombucha products, particularly at the end of the fermentation process, has also been previously reported. This phenomenon is attributed to the fact that the hydrolytic enzymes produced from the microbial community in kombucha SCOBY can also degrade or convert the polyphenols in the raw materials and kombucha products into other metabolites, such as caffeine, which can be utilized as a nitrogen source for microorganisms in kombucha SCOBY [14,72,77,78]. Moreover, the polymerization of small polyphenol compounds into high-molecular-weight complexes can also limit the detection of these beneficial components in kombucha products [79].

**Fig. 5:**
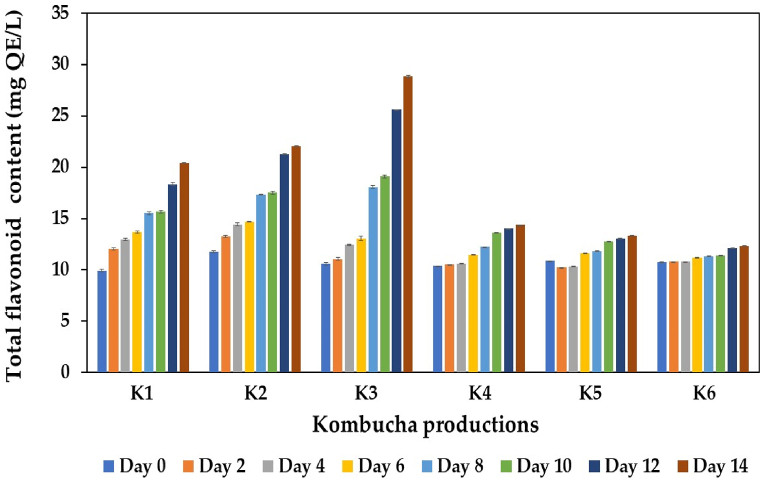
Changes in TFC during kombucha production from black tea alone or with IGH fusions. K1 is black tea kombucha, while K2 to K6 are black tea kombuchas supplemented with 10 %, 20 %, 30 %, 40 %, and 50 % IGH, respectively.

Tea and Indian gooseberry fruits are also known as potential sources of flavonoids, which are significant beneficial constituents that confer benefits to human health due to their antioxidant and antimicrobial properties [52,54,80]. In this study, the TFCs of black tea kombucha (K1) and kombucha produced from black tea and IGH fusions (K2−K6) were evaluated during fermentation, and the results are shown in Fig. 5. The initial TFC of black tea kombucha (K1) was 9.92 mg QE/L, while those of the kombuchas produced from black tea and IGH fusions (K2−K6) ranged from 10.38 to 11.78 mg QE/L, which was slightly higher than that of the control kombucha. As shown in the present study, the TFC significantly increased during the fermentation of kombucha, particularly in black tea kombucha and that supplemented with 10 % IGH (K2) and 20 % IGH (K3), possibly owing to the degradation of polyphenols in black tea and IGH into small molecules by enzymes secreted from the microbial community in kombucha SCOBY. These findings aligned with those of Zubaidah et al. [81], Vitas et al. [82], and Klawpiyapamornkun et al. [6,42]. The highest TFC was detected in K3 kombucha, with a value of 28.82 mg QE/L, followed by K2 kombucha (22.06 mg QE/L). Only slight increases in the TFC of kombucha samples containing 30 %, 40 %, or 50 % IGH were detected. A high proportion of IGH results in highly acidic kombucha products, which may affect the activity of enzymes involved in the conversion or degradation of flavonoids or prevent extractable reduction and esterification reactions during fermentation [83].

Kombucha is a slightly sweet and carbonated acidic beverage. It is classified as a low- or nonalcoholic beverage since its alcohol content is less than 0.5 % (v/v) [13]. As shown in Table 2, all of the fermented kombucha products derived in this work contained low concentrations of ethanol, ranging from 0.047 to 0.137 % (v/v), suggesting that these products could be used as functional low-grade alcoholic beverages. It should be noted that kombucha samples containing Indian gooseberry (K2 to K6) generally exhibited higher ethanol concentrations compared to the black tea kombucha (K1), although this relationship was not linear with increasing Indian gooseberry content. Interestingly, despite these differences in ethanol content, all samples showed similar initial sugar levels and retained high residual sugar concentrations, suggesting that ethanol production in this context is not solely dependent on sugar consumption. The addition of Indian gooseberry likely altered the nutrient profile and acidity of the fermentation medium, potentially influencing yeast activity and ethanol production. Compounds present in Indian gooseberry, such as amino acids, vitamins, or trace elements, may modulate the activity of alcohol dehydrogenase or other enzymes involved in ethanol metabolism. Alternatively, the altered nutrient profile could favor the growth of certain ethanol-producing microorganisms within the SCOBY community. These findings highlight the complex nature of kombucha fermentation and underscore the need for further research to elucidate the precise mechanisms by which Indian gooseberry addition influences ethanol production and acidity in kombucha. Such insights could have implications for both the sensory properties and potential health effects of novel kombucha formulations. Notably, an increase in the ethanol content of kombucha beverages has also been reported in several other works using different alternative ingredients, such as fruit juices [84] and pineapple peels and cores [7], to supplement black or green teas.

**Table 2:** Ethanol and total acidity of black tea kombucha and fusions of black tea and IGH kombuchas.

Kombucha sample	Ethanol concentration (% v/v)	Total acidity (g/L)
K1	0.047 ± 0.01^f^	6.00 ± 1.02^e^
K2	0.124 ± 0.00 ^b^	7.20 ± 1.05 ^d^
K3	0.103 ± 0.01 ^d^	8.10 ± 0.50 ^d^
K4	0.069 ± 0.00^e^	15.00 ± 1.00^c^
K5	0.137 ± 0.01^a^	21.00 ± 1.04^a^
K6	0.109 ± 0.01^c^	18.00 ± 1.09 ^b^

Means±SDs following different letters within a column were significantly different based on DMRT analysis at *p* < 0.05. K1 is black tea kombucha, while K2 to K6 are black tea kombuchas supplemented with 10 %, 20 %, 30 %, 40 %, and 50 % IGH, respectively.

The total acidity of the black tea kombucha (K1) was 6.0 g/L. Like the ethanol content, supplementing black tea kombucha with IGH significantly increased the total acidity of the finished products, yielding a content of 7.20–21.00 g/L. A high proportion of IGH (30−50 %) yielded remarkably greater total acidity than did IGH supplementation at a low concentration. Generally, several organic acids are produced through the metabolic activity of microbial components in kombucha SCOBY and accumulate in kombucha beverages during fermentation. The production and accumulation of such organic acids not only affect the aroma and flavor but also increase the total acidity of kombucha products [4,5]. The increase in the total acidity of kombucha products prepared with black tea and IGH fusions might be attributed to the organic acids in IGH, as these products contain high concentrations of ascorbic acid [33,[56], [57], [58], [59]]. This finding was similar to that of other kombucha products made from the fusion of tea and other alternative ingredients, such as fruit juices and pineapple peels and cores [7,84].

It is worth noting that high total acidity in kombucha beverages may potentially contribute to acidosis, which can adversely affect human health [85,86]. To mitigate this risk, reducing the acidity of kombucha beverages before consumption may be necessary. This can be achieved through dilution with water or by blending with less acidic ingredients. For instance, rooibos tea (*Aspalathus linearis*), an herbal tea with low tannin content and relatively low acidity, can complement kombucha flavors while helping to moderate the overall acidity of the final product [87].

### 3.3 Color of kombucha beverages

Kombucha color is also an important parameter for determining the visual quality of beverages [88]. In general, the color of kombucha products differs depending on the type of tea used. For instance, black tea kombucha appears brown with amber and yellow tones [89]. In this study, the color of the kombucha beverages was determined using the L∗, a∗, and b∗ values. As shown in Table 3, the L∗ values of all kombucha samples (K1 to K6) were not statistically significantly different from each other. However, a trend was observed where the black tea kombucha (K1) and kombucha tea supplemented with 10 % (v/v) IGH (K2) had numerically similar L∗ values, while the kombucha teas supplemented with higher proportions of IGH (K3 to K6) showed slightly higher L∗ values. This subtle trend suggests that increasing IGH concentration may contribute to a slightly lighter appearance in the kombucha products, although these differences were not statistically significant in the current study. Analysis of the a∗ and b∗ values revealed differences in color characteristics among the kombucha samples. The control kombucha (K1) and the kombucha supplemented with 10 % IGH (K2) showed no statistical difference in a∗ and b∗ values, indicating comparable color profiles. However, kombuchas with higher IGH concentrations (K3 to K6) exhibited significantly lower a∗ and b∗ values compared to K1. These findings suggest that the control kombucha (K1) and the 10 % IGH kombucha (K2) shared similar brown hues with amber and yellowish tones. In contrast, kombuchas produced with higher proportions of IGH fusion (K3 to K6) displayed less intense amber and yellow tones, resulting in a lighter color profile. This color shift may be attributed to the increasing concentration of IGH in the kombucha formulations. These results are similar to previous studies in which pineapple peels and cores were used as alternative ingredients to supplement black tea kombucha [7].

**Table 3:** L∗, a∗, and b∗ color values of black tea kombucha and fusions of black tea and IGH kombuchas.

Kombucha sample	L∗	a∗	b∗
K1	84.29 ± 0.50^a^	2.66 ± 0.13^a^	42.02 ± 0.71^a^
K2	84.09 ± 0.90^a^	1.07 ± 0.17 ^ab^	39.96 ± 1.64 ^b^
K3	86.75 ± 0.10^a^	−0.37 ± 0.06 ^b^	39.35 ± 0.85 ^bc^
K4	85.91 ± 0.42^a^	0.14 ± 0.02 ^b^	37.77 ± 0.93^c^
K5	86.33 ± 0.41^a^	−0.35 ± 0.09 ^b^	34.89 ± 0.19 ^d^
K6	86.76 ± 0.63^a^	0.59 ± 0.08 ^b^	39.59 ± 0.59 ^b^

Means±SDs following different letters within a column were significantly different based on DMRT analysis at *p* < 0.05. K1 is black tea kombucha, while K2 to K6 are black tea kombuchas supplemented with 10 %, 20 %, 30 %, 40 %, and 50 % IGH, respectively.

### 3.4 Volatile compounds in kombucha beverages

Several volatile compounds have been identified in kombucha beverages, and their types and concentrations vary depending on the raw materials, fermentation conditions, and microbial compositions in kombucha SCOBY. As demonstrated by de Melo et al. [90] and Tejedor-Calvo and Morales [44], various volatile compounds, such as esters, carboxylic acids, phenols, alcohols, aldehydes, alkanes, ketones, amines, hydrocarbon lactones, and terpenes, have been reported in kombucha products. In this study, a total of 27 volatile compounds were detected in kombucha products via SPME−GC‒MS analysis. These volatile compounds included 7 esters, 6 alcohols, 7 carboxylic acids, 2 phenols, 1 aldehyde, 2 ketones, and 2 benzenes (Table 4). Alkanes, amines, hydrocarbon lactones, and terpenes were not detected in the kombucha products obtained in this work. Sixteen volatile compounds were present in the control kombucha (K1), and five of them, including ethyl hexanoate, ethyl 2-(5-methyl-5-vinyltetrahydrofuran-2-yl)propan-2-yl carbonate, 2,3-butanediol, acetoin, and 4-cyclopentene-1,3-dione, were present only in the control and not in the kombucha beverages prepared with black tea and IGH fusions. In contrast, several volatile compounds, such as ethyl dodecanoate, 2-ethylhexyl salicylate, 2-methyl-1-butanol, β-linalool, phenylethyl alcohol, trans-nerolidol, octanoic acid, ethyl cinnamate, dodecanoic acid, 4-ethylphenol, and benzaldehyde, were not detected in the control kombucha but were present in the kombucha beverages prepared with black tea and IGH fusions, specifically at a high proportion of IGH. These volatile compounds may be introduced into kombucha products during fermentation from raw material, similar to the findings of Phung et al. [7] and Klawpiyapamornkun et al. [6]. Notably, the levels of some volatile compounds, such as ethyl decanoate, phenethyl acetate, ethanol, isobutyric acid, and n-decanoic acid, decreased during fermentation, while those of ethyl acetate and 4-ethyl-2-methoxyphenol increased. Changes in volatile content during fermentation might be attributed to the microbial community metabolic activity in kombucha SCOBY as well as chemical reactions during the incubation of kombucha products.

**Table 4:** Volatile compounds in black tea kombucha and fusions of black tea and IGH kombuchas.

Volatile compound	Rt^a^ (min)	% area∗∗					
		K1	K2	K3	K4	K5	K6
**Esters**							
Ethyl acetate	6.1	1.61	4.31	4.58	2.73	3.57	3.45
Ethyl hexanoate	17.6	0.42	ND	ND	ND	ND	ND
Ethyl 2-(5-methyl-5-vinyltetrahydrofuran-2-yl)propan-2-yl carbonate	27.9	2.58	ND	ND	ND	ND	ND
Ethyl decanoate	34.7	3.99	3.44	2.65	3.52	3.58	3.94
Phenethyl acetate	40.9	2.54	2.45	1.52	2.47	2.69	2.23
Ethyl dodecanoate	42.3	ND	ND	ND	0.63	1.30	1.58
2-Ethylhexyl salicylate	55.6	ND	3.29	1.63	1.07	0.92	0.80
***Alcohols***							
Ethanol	7.2	3.76	3.46	2.77	2.75	1.89	3.19
2-methyl-1-butanol	16.4	ND	ND	ND	0.44	0.67	1.04
2,3-Butanediol	30.6	1.88	ND	ND	ND	ND	ND
β-Linalool	30.9	ND	ND	ND	ND	0.61	0.53
Phenylethyl alcohol	44.2	ND	ND	ND	ND	ND	7.67
trans-Nerolidol	48.8	ND	1.40	1.47	1.44	2.11	0.88
***Carboxylic acids***							
Acetic acid	26.4	45.42	41.79	41.99	50.59	36.73	38.27
Isobutyric acid	31.4	2.50	1.98	1.93	0.99	0.69	0.73
2-Methylhexanoic acid	35.4	7.53	11.33	11.09	6.47	5.17	4.27
Octanoic acid	49.2	ND	7.02	7.79	5.42	6.69	5.64
Ethyl cinnamate	51.2	ND	ND	1.38	2.06	6.10	7.58
n-Decanoic acid	54.9	10.42	5.23	6.01	6.05	9.67	7.58
Dodecanoic acid	59.4	ND	ND	ND	ND	3.30	1.91
***Phenols***							
4-Ethyl-2-methoxyphenol	48.0	1.97	2.89	3.23	2.29	6.34	2.16
4-Ethylphenol	52.2	ND	5.36	5.11	3.36	2.88	2.50
***Aldehyde***							
Benzaldehyde	29.5	ND	3.31	2.58	2.49	2.54	2.67
***Ketone***							
Acetoin	19.6	1.99	ND	ND	ND	ND	ND
4-Cyclopentene-1,3-dione	31.9	1.31	ND	ND	ND	ND	ND
***Benzenes***							
Styrene	18.2	0.60	0.31	0.20	0.21	0.17	0.15
Naphthalene	38.0	2.55	1.63	0.95	1.21	1.14	0.99

a Rt means retention time. ∗∗ Based on SPME−GC‒MS analysis, the number is expressed as a percentage of the area where all the compounds were detected in the kombucha beverages. ND means not detected. K1 is black tea kombucha, while K2 to K6 are black tea kombuchas supplemented with 10 %, 20 %, 30 %, 40 %, and 50 % IGH, respectively.

**Fig. 6:**
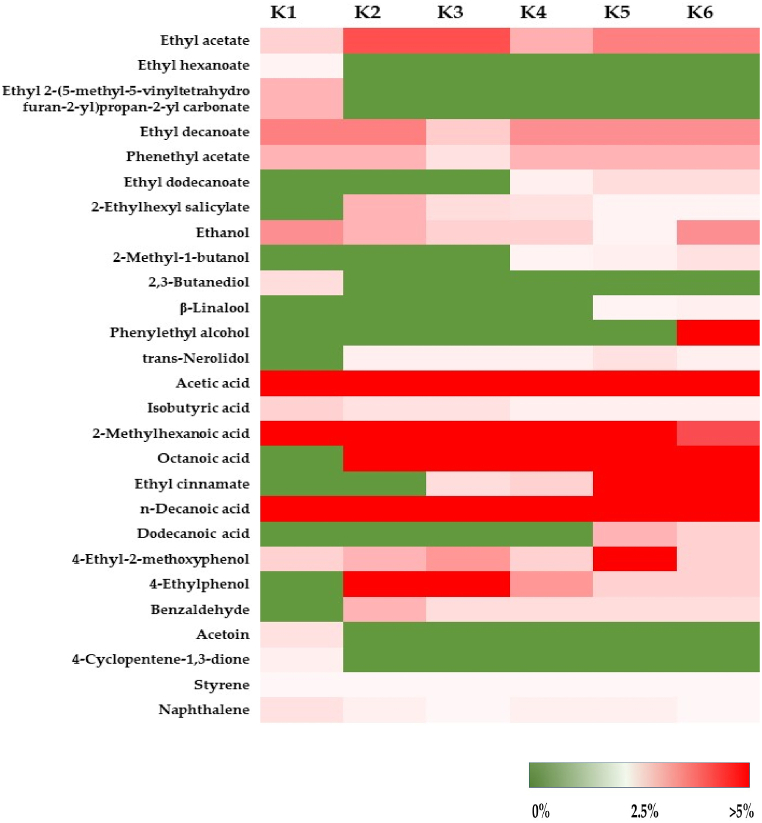
Heatmap profile of volatile compounds in kombucha beverages prepared with black tea and fusions of black tea and IGH. The colors represent the relative percentage of the total volatile compounds detected in the kombucha products and range from green (0 %) to light pink (2.5 %) and red (>5 %). K1 is black tea kombucha, while K2 to K6 are black tea kombuchas supplemented with 10 %, 20 %, 30 %, 40 %, and 50 % IGH, respectively.

As shown in Fig. 6, acetic acid, 2-methylhexanoic acid, and n-decanoic acid, which are carboxylic acids, were the predominant volatile compounds found in all of the kombucha beverages and had relatively high contents compared to the other compounds. These volatile acids contributed mainly to the acidic aroma, particularly acetic acid, which confers a vinegar-like flavor to the kombucha beverages. Although different microbes, such as *Blautia* sp., *Faecalibacterium* sp. [91,92], and *Brettanomyces* sp. [93,94], can produce acetic acid, its production is mainly associated with acetic acid bacteria (AAB). Ethanol serves as a precursor for acetic acid production by AAB. During kombucha fermentation, several species of AAB are known as acetic acid producers, and the predominant species include *Komagataeibacter xylinus*, *K. rhaeticus*, and *K. intermedius* [4,7,44]. 2-Methylhexanoic acid is a medium-chain fatty acid that combines cheesy, creamy, and fruity flavors in kombucha beverages. This volatile acid is rarely found in kombucha beverages except in the spirit [76,95]. However, a recent study by Phung et al. [7] noted that kombucha produced from fusions of black tea and pineapple peels and cores contained this volatile acid in the finished products. Decanoic or capric acid is another medium-chain and saturated fatty acid. This volatile acid is naturally found in plant oils and milk and has a rancid and fatty aroma. Although capric acid has a negative impact on the organoleptic properties of alcoholic beverages, it is used in the manufacturing of esters for artificial fruit flavors and perfumes [96].

The most predominant volatile esters detected in all the kombucha beverages included ethyl acetate, ethyl decanoate, and phenethyl acetate, which are commonly present in several alcoholic beverages, such as wines, fruit distillates, and spirits [76,96,97], as well as vinegar [98]. Ethyl acetate is a short-chain organic ester formed between acetic acid and ethanol. This compound contributes a sweet smell and fruity aroma, similar to a pineapple flavor [44]. Ethyl decanoate, or capric acid ethyl ester, is a fatty acid ester. This compound is considered a highly positive flavor attribute of the young wine aroma and introduces floral and fruity flavor notes. Other beverages, such as distilled coffee and peach spirits, also contain high amounts of this fatty acid ester [76,96]. This volatile ester has also been detected in kombucha beverages prepared with fusions of black tea and pineapple peels and cores [7]. Phenethyl acetate is an acetate ester resulting from the condensation of acetic acid and phenethyl alcohol. This compound is commonly found in several fruits and vegetables, such as melon, pineapple, grape, olive, and lettuce plants, and has a rose and honey scent and a raspberry-like taste. Phenethyl acetate is widely used as a fragrance ingredient in many products, such as decorative cosmetics, fine fragrances, shampoos, toilet soaps, and noncosmetic products, such as household cleaners and detergents [99]. This compound is reported to be found in many products, such as cheddar cheeses, wines, brandies, other grape-derived alcoholic beverages, and kombucha beverages [7,76].

Notably, aromatic volatile organic compounds such as styrene and naphthalene were also detected at relatively low levels in the kombucha beverages. To our knowledge, these two volatile organic compounds have never been reported in kombucha products. Styrene has been reported to be naturally found in a variety of products, such as fruits, vegetables, nuts, beverages, and meats, and to contribute to a sweet or floral aroma [100]. Naphthalene, the smallest and simplest member of the polycyclic aromatic hydrocarbon family, has also been reported in foods, especially those of plant origin, such as cereal products, vegetables, and fruits. This compound can be formed during food processing involving thermal treatment, specifically under relatively high-temperature conditions. A study by Cao et al. [101] demonstrated that fortified soy beverages contained this aromatic volatile compound at 0.66 ng/g. Typically, this compound has a very pungent odor, which may negatively impact the sensory quality of alcoholic beverages.

### 3.5 Antioxidant activity of kombucha beverages

**Fig. 7:**
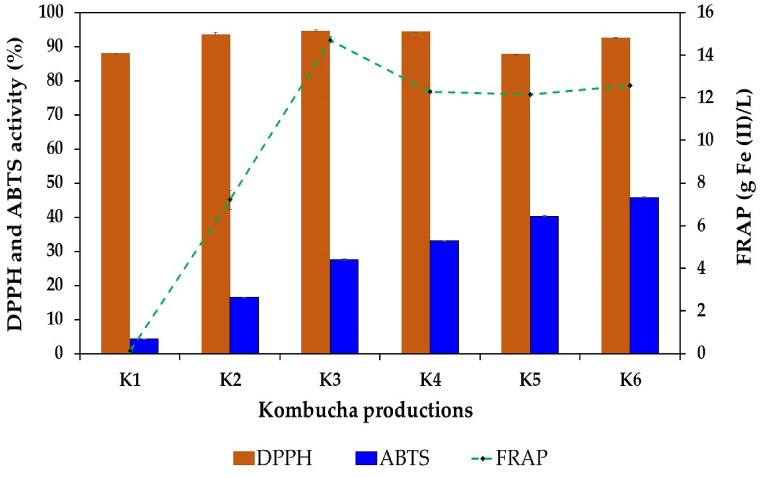
Antioxidant activity of kombucha products prepared with black tea alone or with IGH fusions. K1 is black tea kombucha, while K2 to K6 are black tea kombuchas supplemented with 10 %, 20 %, 30 %, 40 %, and 50 % IGH, respectively.

Kombucha is known to possess potent antioxidant capacity since it contains several beneficial substances, including polyphenols, flavonoids, organic acids, vitamins, and trace elements; these substances come from raw materials and are also generated during fermentation by the metabolic activity of the microbial community in kombucha SCOBY [[5], [6], [7], [8]]. Because different bioactive compounds use different mechanisms to react with free radicals, several antioxidant detection assays have been developed. DPPH, ABTS, and FRAP assays are the most commonly used among the different detection techniques, especially for food and beverage products [6,7,24,42]. As shown in Fig. 7, the kombuchas produced with black tea and IGH fusions displayed overall greater antioxidant activity than the control black tea kombucha. The DPPH assay revealed that the kombucha beverages prepared with black tea and IGH fusions exhibited slightly greater values than the control kombucha beverages. The highest DPPH activity was 94.67 % in K3 kombucha, followed by 94.34 % in K4 kombucha, while that of the control was 87.98 %. Regarding the ABTS and FRAP activities, the kombuchas produced from black tea and IGH fusions displayed significantly greater values than the control kombucha beverages. The highest ABTS activity was detected in K6 kombucha (45.79 %), whereas the highest FRAP activity was observed in K3 kombucha (14.71 g Fe(II)/L). The high antioxidant activity of kombucha beverages prepared with black tea and IGH fusions may be attributed to the high polyphenol, flavonoid, organic acid, and vitamin contents extracted from black tea and IGH. Like in other works, supplementing kombucha tea with fruit juice, fruit waste, olive leaf, and honey materials also enhanced the antioxidant activity of kombucha beverages [7,21,50,81,102].

### 3.6 Antimicrobial activity of kombucha beverages

Based on the literature review, kombucha is known to possess antimicrobial activity against several microorganisms, specifically pathogenic bacteria and yeasts [7,42,103]. The antimicrobial properties of kombucha beverages are closely correlated with many beneficial compounds, especially polyphenols, flavonoids, organic acids, alcohols, and volatile organic compounds, which are liberated from raw materials or generated during the kombucha fermentation process through the metabolic activity of the microbial community in the kombucha SCOBY. Typically, the antimicrobial effect of a fungus against microbes depends on the type and concentration of beneficial compounds in the kombucha product. Several previous studies reported that supplementing kombucha tea with alternative ingredients, such as lemon balm materials [104] and pineapple peels and cores [7], improved not only the organoleptic properties but also the antimicrobial activity of kombucha products. As shown in this study, supplementing black tea kombucha with IGH, specifically at high proportions ranging from 30 % to 50 % (v/v), markedly inhibited the growth of almost all the tested microorganisms, except for the gram-positive bacterium *B. cereus* (Table 5). The acetic acid used as the positive control in this study also exhibited broad-spectrum inhibition of the growth of all the microorganisms tested. As previously shown in this study, kombucha beverages prepared with black tea and IGH fusions exhibited significantly greater total acidity and TPC than the control kombucha beverages. Thus, the high antimicrobial efficacy of black tea and IGH fusion kombuchas against pathogenic microorganisms may be attributed to the organic acid (specifically acetic acid) and polyphenol contents in the kombucha products. Many studies have also shown that the antimicrobial efficiency of kombucha species is closely attributed to the presence of organic acids, especially acetic acid and polyphenols, which are considered the major antimicrobial components of kombucha beverages [14,105,106].

**Table 5:** Antimicrobial activity of black tea and fusions of black tea and IGH kombuchas.

Microbial strain	Diameter of inhibition zone (mm)^a^							
	Ac∗∗	Water	K1	K2	K3	K4	K5	K6
***Gram-negative bacteria***								
*Enterobacter cloaceae*	27.3 ± 2.9	0	0	0	0	0	0	15.3 ± 3.5
*Escherichia coli* (ATCC 25922)	27.0 ± 2.6	0	0	0	0	0	0	16.3 ± 2.1
*Pseudomonas aeruginosa* (ATCC 27853)	26.7 ± 2.5	0	0	0	0	0	13.3 ± 0.6	15.7 ± 2.9
*Salmonella enterica* Enteritidis	25.3 ± 5.5	0	0	0	0	0	13.6 ± 2.6	17.3 ± 2.1
*S. enterica* Typhimurium	22.3 ± 4.9	0	0	0	0	0	0	13.0 ± 1.0
*S. enterica* Typhi (DMST 22842)	30.0 ± 1.0	0	0	0	0	0	14.3 ± 1.5	18.3 ± 2.1
*Shigella dysenteriae* (DMST 1511)	29.7 ± 1.2	0	0	0	0	0	0	15.0 ± 4.6
***Gram-positive bacteria***								
*Bacillus cereus*	24.3 ± 4.5	0	0	0	0	0	0	0
*Listeria monocytogenes* (ATCC 19115)	26.0 ± 2.6	0	0	0	0	0	14.7 ± 1.2	19.7 ± 1.5
*Staphylococcus aureus* (ATCC 25923)	29.3 ± 1.5	0	0	0	0	0	0	13.3 ± 3.0
***Yeast***								
*Candida albicans*	30.3 ± 1.5	0	0	0	0	16.0 ± 1.0	18.3 ± 1.5	19.3 ± 1.5

a Means ± SDs, including a well diameter of 6 mm ∗∗Ac means acetic acid. K1 is black tea kombucha, while K2 to K6 are black tea kombuchas supplemented with 10 %, 20 %, 30 %, 40 %, and 50 % IGH, respectively.

In addition to organic acids and polyphenols, other beneficial compounds, especially alcohols and volatile organic compounds, also contribute to the antimicrobial activity of kombucha products [48,104]. As shown in this study, several volatile compounds, such as phenylethyl alcohol, octanoic acid, ethyl cinnamate, 4-ethylphenol, and benzaldehyde, were detected in kombucha beverages prepared with black tea and IGH fusions but not in control kombucha. These volatile active compounds may also contribute to the antimicrobial efficacy of black tea and IGH fusion kombucha beverages. These findings suggested that IGH is an effective ingredient for enhancing the antimicrobial efficacy of kombucha beverages. Notably, black tea kombucha and kombucha prepared with black tea fusions using 10−20 % (v/v) IGH showed no antimicrobial activity against any of the tested microbes, possibly due to the low concentration of active antimicrobial compounds. These findings are similar to those of Kaewkod et al. [14], Phung et al. [7], and Klawpiyapamornkun et al. [42].

It should be noted in this study that the K5 and K6 kombucha products demonstrated remarkable antimicrobial potential through a broad range of pathogen inhibition. Volatile organic compound analysis revealed three predominant compounds unique to these kombucha products: ethyl dodecanoate, β-linalool, and dodecanoic acid. Previous research has extensively documented the antimicrobial properties of these compounds against various pathogenic microorganisms. β-Linalool has shown effective inhibition of bacterial and fungal growth, with significant antimicrobial activity against *S*. *aureus* and *E*. *coli*. Dodecanoic acid (lauric acid) disrupts bacterial cell membranes, particularly targeting gram-positive bacteria, while ethyl dodecanoate exhibits moderate antimicrobial potential against food-borne pathogens [107,108]. Therefore, based on this information, we hypothesize that the substantial antimicrobial activity observed in K5 and K6 kombucha products may be primarily attributed to the presence of these specific volatile organic compounds.

## 4 Conclusion

Although several alternative raw materials have been used to replace or supplement tea leaves to produce kombucha beverages, supplementing black tea with IGH was shown to be an excellent combination for producing functional kombucha beverages in this study. IGH contains various bioactive components, including polyphenols, flavonoids, vitamins, amino acids, fatty acids, and trace elements, which benefit human health. The TPC, TFC, total acidity, and volatile organic compound content could be improved by supplementing black tea with IGH. Furthermore, compared with those of control kombucha, the antioxidant and antimicrobial activities of kombucha beverages, specifically those produced from a high proportion of IGH, were also significantly greater. Based on the biochemical changes and health benefits, specifically antioxidant activity, supplementing black tea kombucha with 20 % IGH was an excellent combination for functional kombucha production. The results obtained in this study suggested that Indian gooseberry fruit has high potential for use as a supplement to black tea in the production of new alternative functional and unique kombucha beverages.

Regarding organoleptic properties, further studies, such as sensory evaluation and the correlation analysis between volatile organic compounds and the aroma or taste of kombucha products or the dynamic changes of microbial community during the fermentation process, are needed. Furthermore, further studies, specifically the *in vitro* and *in vivo* biological property analysis of kombucha beverages, are also needed to determine the human health effects of these products.

## CRediT authorship contribution statement

**Haruthairat Kitwetcharoen:** Writing – original draft, Methodology, Investigation. **Yupaporn Phannarangsee:** Methodology, Investigation. **Preekamol Klanrit:** Writing – review & editing, Writing – original draft, Validation, Methodology, Formal analysis, Conceptualization. **Sudarat Thanonkeo:** Writing – review & editing, Writing – original draft, Validation, Software, Methodology, Formal analysis, Conceptualization. **Patcharaporn Tippayawat:** Writing – review & editing, Resources, Conceptualization. **Poramaporn Klanrit:** Writing – review & editing, Writing – original draft, Validation, Methodology, Formal analysis, Conceptualization. **Poramate Klanrit:** Writing – review & editing, Writing – original draft, Validation, Methodology, Formal analysis, Conceptualization. **Mamoru Yamada:** Writing – review & editing. **Pornthap Thanonkeo:** Writing – review & editing, Writing – original draft, Visualization, Validation, Software, Resources, Project administration, Methodology, Investigation, Funding acquisition, Formal analysis, Data curation, Conceptualization.

## Data availability statement

Data will be made available on request.

## Funding statement

This research was funded by the National Research Council of Thailand under the research program “Increasing the Value of Fruit Trees to High-value Agriculture.” The financial support was also from the Research and Graduate Studies, Khon Kaen University, through the Research Program of the Fermentation Research Center for Value Added Agricultural Products (FerVAAPs).

## Declaration of competing interest

The authors declare that they have no known competing financial interests or personal relationships that could have appeared to influence the work reported in this paper.
